# Woman at Home

**Published:** 1887-11-12

**Authors:** 


### THE DOCTOR'S PULPIT.

### WOMAN AT HOME.

The principal business of women is domestic management, and for this they are not thoroughly trained. That was the contention of a former paper. Women claim a kind of equality with men, a claim which sense and experience concur in considering reasonable. They have not the same physical vigour as men, but that is merely a matter of degree. When they are trained in physical exercises they approach man in physical vigour, just as a boy does. We do not say a boy should not do the same *kind* of work as a man because his strength is not so great. Neither should we, if we were unprejudiced, say that women ought not to do the same kind of work in those frequent cases where they have energy for it. The recent outcries against women working at the pit-brow and at field labour, were sentimental and foolish. Women, like men, are made stronger by outdoor labour; and there is no more reason why they should be unsexed by it than that men should be morally injured. Similarly, when the argument is taken into the regions of study and business capacity, it is found that women possess the same kind of intelligence and fitness as men. If they differ at all it is almost entirely in degree. They can teach mathematics when they have to do so; they can keep shops and conduct businesses; they can be farmers and lawyers. It is not said that they should; but it is affirmed that their intelligence is of the same order as that of men, and therefore as capable of being trained to high efficiency in any work they may have to do. Is it necessary to train men for their special work in life? It is just as necessary to train women. Do trained men beat untrained men entirely out of the field? In the same way would trained women beat their untrained sisters. Whatever arguments can be advanced in favour of special and thorough training for men, the very same arguments are as convincing in favour of the special and thorough training of women.

If this be not so then one of two things must be granted. Either domestic management is so easy and simple that no special training is needed for it; or women are such born geniuses that they can carry out what seems to men a difficult and complicated business by instinct and intuition. It is probable that some of the more foolish among them are prepared to accept either of these alternatives; but the wiser and more instructed certainly are not.

Before entering more into detail on the question of special education in domestic management, a few words may be permitted on the relative importance of the subject itself. Many women long for a career; they sigh because they are not men—because their lives are so purposeless and inane. Nothing is more natural. A woman who has no career, and who does not desire one, is little better than an imbecile. No words need be wasted in describing her, because she is not worth even so much as half-a-dozen lines of description. But what special career should a woman look to? If she has any special aptitudes and opportunities, let her, without hesitation or fear, follow her aptitudes, and make use of her opportunities. So far as pure science speaks there is no manner of reason why a woman should not follow any career in the world. If she is fit for the woolsack, or for the archiepiscopal throne, or for the presidency of the College of Physicians, let her aim at these exalted positions and reach them if she can. Who is to say her nay? We, at any rate, are fully convinced that women, like men, have the fullest possible right to "a fair field and no favour." Let then those who want to be doctors be doctors; those who love literature, or music, or painting, or teaching, paint and teach and follow music and literature as careers. But these are the exceptions. What is to be done with the majority, that vast number who by force of instinct, habit, aptitude, or circumstances, find a domestic career as naturally marked out for them as the summer bed of the river is for the winter torrent?

What, we ask, is the objection to a domestic career? Is it not intellectual? Is there no heroism about it? Does it make no demand on the practical business capacity? Is there no opportunity for the practice of the highest morality; the most beautiful self-denial; the purest religion? One or two practical tests will answer these questions. Would a man reverence his mother more if she were a painter or writer of books than he does when she is simply the incarnation of home? Would he love and trust his wife more completely if she could plead at the bar or diagnose an internal aneurism? What is it that really constitutes the importance of any given career? Is it the career itself or the way in which it is pursued. May not a Minister of State be a very worthless person; and may not the ploughman who tills his fields be a very noble man? But apart from these considerations, the career of manager of the affairs of the Home is of the first importance. Food is more important than physic, because it nearly concerns an infinitely larger number of persons, and also because it is an absolute necessity, whereas physic is more or less of an accident or a luxury. Do we then believe the management of food and all that it involves to be of greater absolute importance than the management of physic and all that it involves? Certainly! And do we admit the inevitable corollary that housewives as a class are more important than doctors as a class? Unquestionably! And therein lies the weightiness of the whole matter. Domestic management is often despised by women, and relegated to a secondary place by men, not because it is in itself unworthy of honour or deserving only of a second-rate position, but because the men and the women who so regard it either have not the capacity, or have never taken the trouble, to form a just estimate of the subject

The conventional view of the relative importance of men and things is often as far asunder from the true view as gold is from forged notes; and there is no better illustration of this proposition than the low level to which mere housekeeping has sunk among us. We need not remind readers over again of that of which they have so often been reminded before, that the grandmothers of those who are most ready to boast of having had grandmothers, took a very different view. But we may appeal to the supposed superior education of modern times, which should be accompanied by superior judgment. Let domestic management be tried by any standard the reader may select, and it cannot be held as mean or un-

**important. In large establishments, where wealth is abundant and position has to be maintained, its status is admitted by the fact that accomplished and specially trained persons are paid high salaries to do this one thing. But in more modest circumstances, where the means are more limited and where it is absolutely necessary to expend every sixpence in the very wisest way, how much more essential is it that the chancellor of the domestic exchequer shall not only be an accomplished mistress of finance, but a highly skilled administrator in every other department of the domestic state. We express the emphatic conviction that as no lot is so desirable in every way for the average woman as to be the wife of a good and honourable man, so no career is so suitable for her or so well worthy of her best and noblest powers as that of being at the head of his and her own domestic affairs. So far as bodily and mental health are concerned no person whatsoever is more potent for good or for ill than the housewife.**

